# Attenuation of Wnt/β-catenin signaling in patients with Stevens-Johnson syndrome and toxic epidermal necrolysis

**DOI:** 10.7150/ijbs.32331

**Published:** 2020-01-01

**Authors:** Chun-Bing Chen, Wan-Chun Chang, Ming-Ying Wu, Tzu-Yang Kao, Ying-Wen Wang, Chuang Wei Wang, Chi-Ju Chen, Wen-Hung Chung, Shih-Chi Su

**Affiliations:** 1 Department of Dermatology, Drug Hypersensitivity Clinical and Research Center, Chang Gung Memorial Hospitals, Linkou, Taipei, and Keelung, Taiwan.; 2 Whole-Genome Research Core Laboratory of Human Diseases, Chang Gung Memorial Hospital, Keelung, Taiwan.; 3 Chang Gung Immunology Consortium, Chang Gung Memorial Hospital and Chang Gung University, Taoyuan, Taiwan.; 4 College of Medicine, Chang Gung University, Taoyuan, Taiwan.; 5 Graduate Institute of Clinical Medical Sciences, College of Medicine, Chang Gung University, Taoyuan, Taiwan.; 6 Immune-Oncology Center of Excellence, Chang Gung Memorial Hospital, Linkou, Taiwan.; 7 Institute of Microbiology and Immunology, National Yang-Ming University, Taipei, Taiwan.; 8 Department of Dermatology, Xiamen Chang Gung Hospitals, China.

**Keywords:** Stevens-Johnson syndrome (SJS), toxic epidermal necrosis (TEN), cytotoxic T lymphocyte (CTL), Wnt, T cell factor-1 (TCF-1), lymphoid enhancer binding factor 1 (LEF-1).

## Abstract

Stevens-Johnson syndrome (SJS) and toxic epidermal necrosis (TEN) are rare but life-threatening severe cutaneous adverse reactions. Current studies have suggested that the pathobiology of drug-mediated SJS/TEN involves a dysregulation of cellular immunity with overwhelming activation of cytotoxic T lymphocytes. The canonical Wnt signaling pathway plays important roles in T cell development and activation, which may provide potential avenues for alleviating dysregulated immunity in SJS/TEN. In this study, we aimed to assess the implication of Wnt signaling in drug-reactive T cells in SJS/TEN. We showed downregulation of Wnt signaling components, including T cell factor 1 (TCF-1)/lymphoid enhancer binding factor 1 (LEF-1) transcription factors, in SJS/TEN patients, suggesting that canonical Wnt signaling is regulated during cytotoxic T cell responses in SJS/TEN. Further analyses demonstrated that engagement of the T cell receptor by antigen encounter and treatment of a prognostic marker of SJS/TEN, IL-15, *in vitro* led to the downregulation of LEF-1 and TCF-1 expression in CD8+ T cells. Enhancement of Wnt signaling by adding the Wnt activators attenuated *ex vivo* activation of drug-specific T cells from SJS/TEN patients, indicating a functional involvement of Wnt signaling in the pathomechanism of SJS/TEN. These findings provide additional insight into the immunopathogenesis and therapeutic intervention of this devastating condition.

## Introduction

Stevens-Johnson syndrome (SJS) and toxic epidermal necrolysis (TEN) are considered a spectrum of life-threatening severe cutaneous adverse reactions. SJS/TEN share similar clinical presentations like rapidly progressing blistering exanthema and target-like macules. The affected skin specimens typically showed subepidermal blistering, extensive apoptotic keratinocytes and partial- or full-thickness epidermal necrosis and epidermal detachment with a sparse dermal T-cell lymphocyte infiltrates histopathologically 1, 2. Mucosal involvements and skin detachments are also characteristic 3. SJS is defined by the degree of skin detachment less than 10% of body surface area (BSA), while TEN is defined as the area of denuded skin greater than 30% of BSA. Besides, the cases with degree in-between (10-30%) are diagnosed as SJS/TEN overlap.^4^ Though the incidence is relative low 5, 6, they may cause multi-organ failure and significant mortality, with the mortality rate being 10% for patients with SJS, approximately 30% for patients with SJS/TEN overlap and almost 50% for patients with TEN 7, 8.

Although the underlying pathological mechanisms are not fully understood, current pharmacogenomic studies have proposed an association between drug-induced SJS/TEN and human leukocyte antigen (HLA) genes 9-11. Specific HLA molecules may have higher binding affinities for drug antigens and present the drug antigens to T cell receptor (TCR), causing a cascade of T cell activations and aberrant immune responses directed at keratinocytes 3, 12, 13. Though the discovery of the predisposing gene has lowered the incidence, present therapies are mostly empirical 14. The situation points out the fact that there are numerous unanswered questions remain with regard to the immunological and cytotoxic signaling pathways.

The Wnt signaling pathway is evolutionarily conserved and is implicated in a large variety of developmental processes including specification of cell fate and maintenance of stem cell pluripotency 15, 16. In parallel, aberrant Wnt signaling underlies a wide range of pathophysiological conditions 17. This pathway utilizes T cell factor (TCF)/ lymphoid enhancer binding factor (LEF) transcription factors and β-catenin coactivator to achieve balanced regulation of its downstream gene expression. It is well established that several Wnt ligands and their effector proteins are crucial for normal T cell development in thymus 18. Recent studies have also revealed critical requirements for Wnt signal transduction cascade in regulation of mature T cell responses in the circulation 19. Specifically, Wnt pathway orchestrates the generation and persistence of functional memory CD8+ T cells, promotes Th2 differentiation, and suppresses Th17-differentiation of activated CD4+ T cells 20. Activation of β-catenin facilitated CD8+ memory T cell formation, with enhanced protective capacity and extended survival of CD4+CD25+ regulatory T cells (Tregs) 21.

The cytotoxic CD8+ T cells, that are vital in defense against pathogens and non-self antigens (e.g. medication in adverse drug reactions), remain the key player in the patho-mechanism of SJS/TEN. Activation of drug-specific naive CD8+ T cells requires TCR stimulation, co-stimulation, and pro-inflammatory cytokines from antigen-presenting cells and other innate immune cells.^12^ Activated T cells then undergo massive clonal expansion and are equipped with cytokines such as interferon-γ and cytolytic molecules, including granzyme B, perforin, and granulysin.^14^ Yet, only a small portion of activated cells further differentiates into memory CD8+ T cells, which subsequently contribute to the devastating condition in SJS/TEN, a delayed-type hypersensitivity reaction while re-exposed to the same culprit drug. A role of Wnt signaling pathway in promoting generation of memory CD8+ T cells has been demonstrated based on the observation that antigen-primed human memory CD8+ T cells expressed lower TCF-1 and LEF-1, two downstream effectors of Wnt signal, than did naive T cells.^22^ Consistently, constitutive activation of the Wnt signaling pathway was found to reduce effector CD8+ T cell expansion.^23^, ^24^ Another recent study confirmed these findings and further demonstrated that forced expression of stabilized β-catenin in naive T cells interfered with proximal TCR signaling.^25^ These data suggest that modulating the activity of Wnt pathway may be a potential niche to mitigate dysregulated immunity and treat T cell-mediated diseases, such as SJS/TEN. However, little is known about the role of Wnt signaling pathway in the pathobiology of SJS/TEN. In this study, we assessed the implication of Wnt signaling in drug-reactive T cells and also determined whether the Wnt signaling pathway is functionally associated with the disease activity in SJS/TEN.

## Method

### Subject recruitment and sample collection

Patients who fulfilled the consensus diagnostic criteria for SJS/TEN were enrolled at the Chang Gung Memorial Hospital Health System. All cases were evaluated by at least two dermatologists, who reviewed all available photographs, histological data, and clinical information, including type of cutaneous reactions, date of onset, and drug history, dosage, and duration. Phenotypes were clinically assessed using the diagnostic criteria established.^26^ Plasma or serum samples, and PBMC of patients were obtained from whole blood samples as well as blister cells within 2 days after admission(1-7 days after the index day) at the active stage of disease course(before systemic immunomodulatory or immunosuppressive treatment) and after complete skin reepithelization at recovered stage. Blister fluid was collected by puncture of several blisters at the active stage while applicable. In addition to SJS/TEN, blood samples from burn injury patients (n=11) and heathy donors (n=11), and blister fluid from burn injury patients (n=11) were used as control groups. Informed consent was obtained from the participants.

### Quantitative RT-PCR analysis

Total RNA was extracted from cells indicated by TRIzol reagent (Invitrogen, Carlsbad, CA) and checked for quality by the Bioanalyzer 2100 system (Agilent Technologies, CA). An AccuScript High Fidelity 1st Strand cDNA Synthesis Kit (Stratagene) was used to prepare cDNA. Primers were designed using Beacon Designer software. Reactions were analyzed on a Bio-Rad iCycler iQ Multicolor Real-Time PCR Detection system using iQ SYBR Green Supermix (Bio-Rad, Hercules, CA). Each real-time PCR reaction contained 0.5 ng/ul of cDNA and 400 nM of each primer in a 25-ul reaction volume. The reaction was initiated at 94°C for 1.5 min, followed by 40 two-step amplification cycles consisting of 15 s of denaturation at 95°C and 45 s of annealing/elongation at 60°C. A final dissociation stage generated a melting curve for verification of amplicon specificity. Assays were performed in triplicate.

### FACS staining

Cells were subject to methanol permeabilization before examining intracellular expression of LEF1 and TCF-1 within CD8+ T cells. LEF1 and TCF-1 were detected by indirect staining using pre-titrated mAbs REMB6 (Oncogene Research Products) and 7H3 (Upstate Biotechnology), respectively. Briefly, 2 ug of primary mAb were added to 10^6^ cells and incubated for 1 h at room temperature. After washing, this is followed by staining with corresponding 2^nd^ Ab (DakoCytomation) for 1 h at room temperature. Cells were subsequently washed in blocking buffer before surface staining with directly conjugated mAbs specific for CD56 or CD8 (BD Biosciences). The fraction of TCF1^high^ and LEF1^high^ in CD8 T cell subsets were further analyzed and compared between the active stage and the recovery stage in 4 SJS/TEN patients. Furthermore, to illustrate the restriction of stimulation by specific offending drugs, we compared the expression of LEF1 and TCF-1 within CD8+ T cells among groups stimulated by culprit drugs (including 5 SJS/TEN cases: 2 carbamazepine, 1 lamo*trigine, 1 oxcarbazepine, and 1 moxifloxacin-induced SJS/TEN cases)* and tolerant drugs (including phenytoin, clonazepam, and cefazolin).

### Lymphocyte activation test (LAT)

PBMCs (10^6^/ml), obtained from the heparinized blood by density centrifugation on Ficoll-Hypaque, were cultured in RPMI1640 medium supplemented with heat-inactivated AB-serum, glutamine, antibiotic-antimycotic solution and nonessential amino acids. All cultures were performed in triplicate in 96-well plates. Stimuli are the culprit drugs (including 1 carbamazepine and 1 ketoprofen-induced SJS/TEN cases) and the pan T-cell mitogen phytohaemagglutinin (PHA) as a positive control. After 7 days, the stimulation index (SI) was calculated by the level of secreted granulysin of stimulated to unstimulated cultures. The levels of granulysin were determined by home-made ELISA as described previously, 27 whose sensitivity for granulysin is 2.5 ng/mL.

### Evaluation of the circulating Wnt agonists and antagonists

Concentrations of DKK1, SOST, WIF1, and Wnt3a in sera or blister fluid were determined by commercial ELISA (R&D systems, Minneapolis, Minnesota). All samples will be analyzed in triplicate.

### Statistical analyses

Differences in the levels of biomarkers or cell responses between groups were evaluated by the Student's t-test. The paired-sample t-test was applied in the experiments where samples are analyzed in different time points. A *p* value<0.05 was considered significant. The data were processed by using SAS statistical software (Version 9.1, 2005; SAS Institute Inc., Cary, NC).

## Result

### Study subjects

In this study, serum samples from total 25 SJS/TEN patients were used to explore the potential role of Wnt pathway in SJS/TEN. All the cases were collected at the Chang Gung Memorial Hospital Health System which received referral patients from the Taiwan Severe Cutaneous Adverse Reaction Consortium across Taiwan. The distribution of demographic characteristics, phenotypes, and underlying conditions, and causative drugs among patients and controls are shown in Table 1 and Supplement Table 1, respectively. The mean age of the study group is 52.5±18.7 years. There are 7 cases present with SJS, 6 with SJS/TEN overlap, and 12 with TEN. In average, these patients have erythema covered more than half of the total body surface area while blisters or detachments involved 30% of the total body surface area (56.5±26.7 and 30.9±22.9, respectively). The mortality rate is 20.0%. For mucosal involvement, the results show a hundred percent of oral ulcers clinically, followed by genital ulcers and ocular involvements. The most common complications are hepatitis and gastrointestinal bleeding (n=6, 24.0%). The presence of atypical lymphocytes in blood draw was observed in 64.0% of the patients, and 24.0% of the patients have eosinophilia. 16.0% and 36.0% of the patients have leukocytosis and leukopenia respectively while suffer from hypersensitivity reactions. In addition, 28.0% of the patients have thrombocytopenia. Fever episode is also a common presentation and affects more than half of the study group (n=13, 42.0%). Three patients suffered from permanent corneal damage and visual impairments. As for the culprit drugs that these patients used, the offending drugs are mainly anticonvulsant agents (n=8), antibiotics (n=7) and allopurinol (n=4).

### Aberrant expression of Wnt signaling components in PBMCs and blister cells from SJS/TEN patients as well as drug-activated T cells from SJS/TEN patients

**Figure 1:**
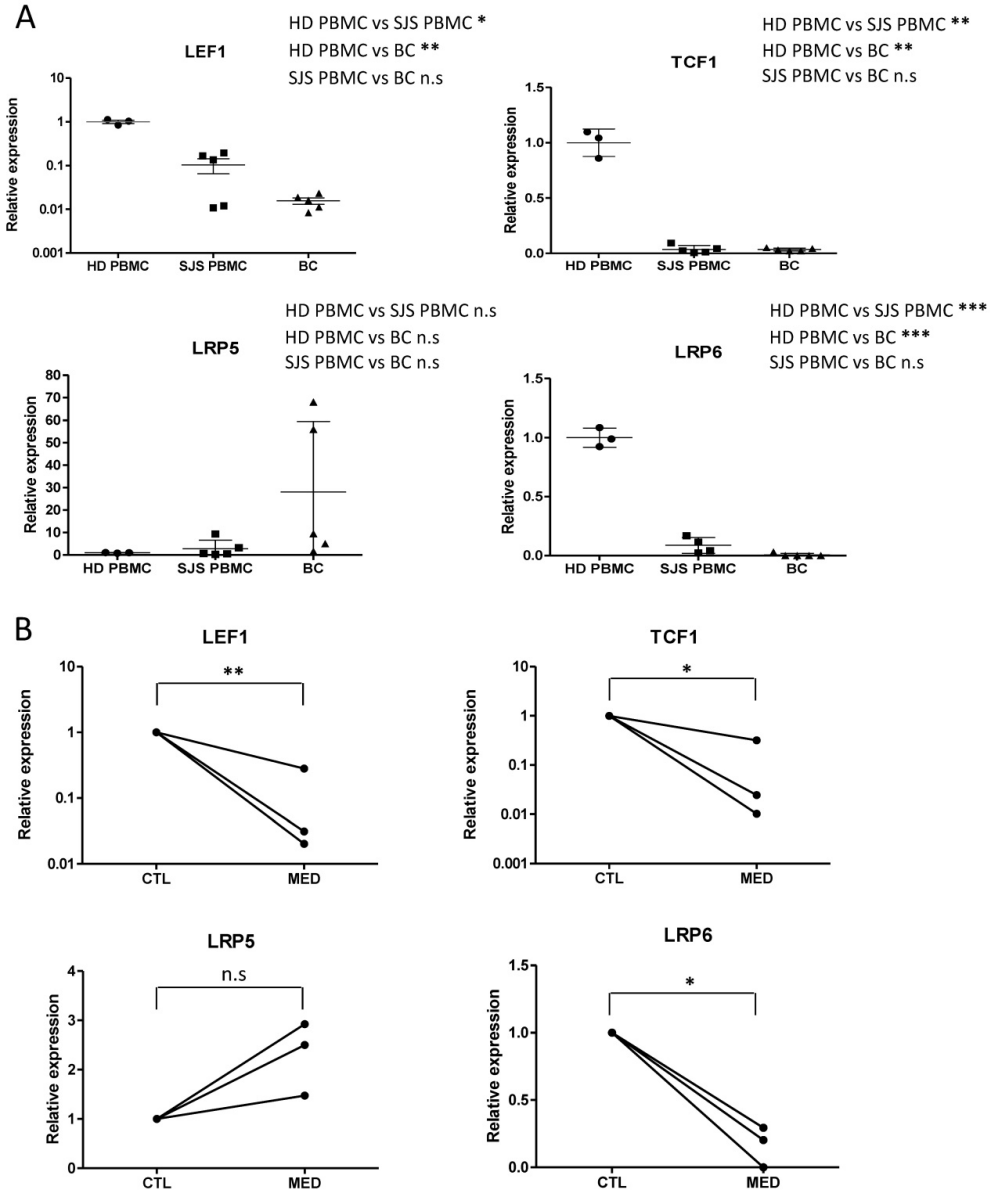
** Expression of Wnt signaling components is regulated in SJS/TEN and drug-specific T cell activation.** (A) Gene expression analyses of Wnt signaling components in blister cells (BCs) and PBMCs of subjects with SJS/TEN. mRNA expression of LEF1, TCF1, LRP5, and LRP6 was determined by real-time PCR. The respective mRNA levels were compared among the blister cells (n=5) and PBMCs (n=5) from subjects with SJS/TEN at active stage and control PBMCs from healthy donors (HD) (n=3). (B) Gene expression of Wnt signaling components in drug-activated T cells from SJS/TEN patients. PBMCs obtained from three SJS patients at active stage were cultured in RPMI1640 medium and stimulated without (Control) and with the culprit drugs (MED), Solaxin, carbamazepine, *and vancomycin, respectively*. After 7 days, the mRNA expression of LEF1, TCF1, LRP5, and LRP6 in drug-sensitive T cells, which were determined with the stimulation index (SI, calculated by the level of secreted granulysin of stimulated to unstimulated cultures) greater than 2, was measured by real-time PCR. *p <0.05; ** p <0.01; ***p <0.001; n.s, not significant; two-sided Student's t-test.

Mounting evidence has shown that Wnt signaling pathway is involved in the regulation of mature T cells 22, 28-30. Upon the activation of effector T cells, components of the Wnt signaling pathway, such as active form of β-catenin (dephosphorylated state), LEF1, TCF1, lipoprotein receptor-related protein-5 (LRP5, Wnt coreceptor), LRP6 (Wnt coreceptor), are differentially expressed. To determine the putative fluctuations in Wnt signaling during the progression of SJS/TEN, expression levels of genes related to Wnt signaling in T cell function, including LEF1, TCF1, LRP5, and LRP6, were assessed in blister cells from SJS/TEN patients and compared with that in PBMCs from SJS/TEN patients and from normal subjects. We showed that a decrease in gene expression for LEF1, TCF1, and LRP6 was detected in SJS/TEN as compared with normal subjects (Figure 1A). Further, we examined the expression of these genes in drug-activated T lymphocytes from SJS/TEN patients. Similar results were obtained in T lymphocytes from SJS patients who were exposed to the causative drug (Figure 1B). However, such downregulation was not observed for LRP5 in PBMCs, blister cells, or drug-activated T lymphocytes from SJS/TEN.

### TCF1 and LEF1 protein is downregulated in CD8+ T cells at the active stage of SJS/TEN

**Figure 2:**
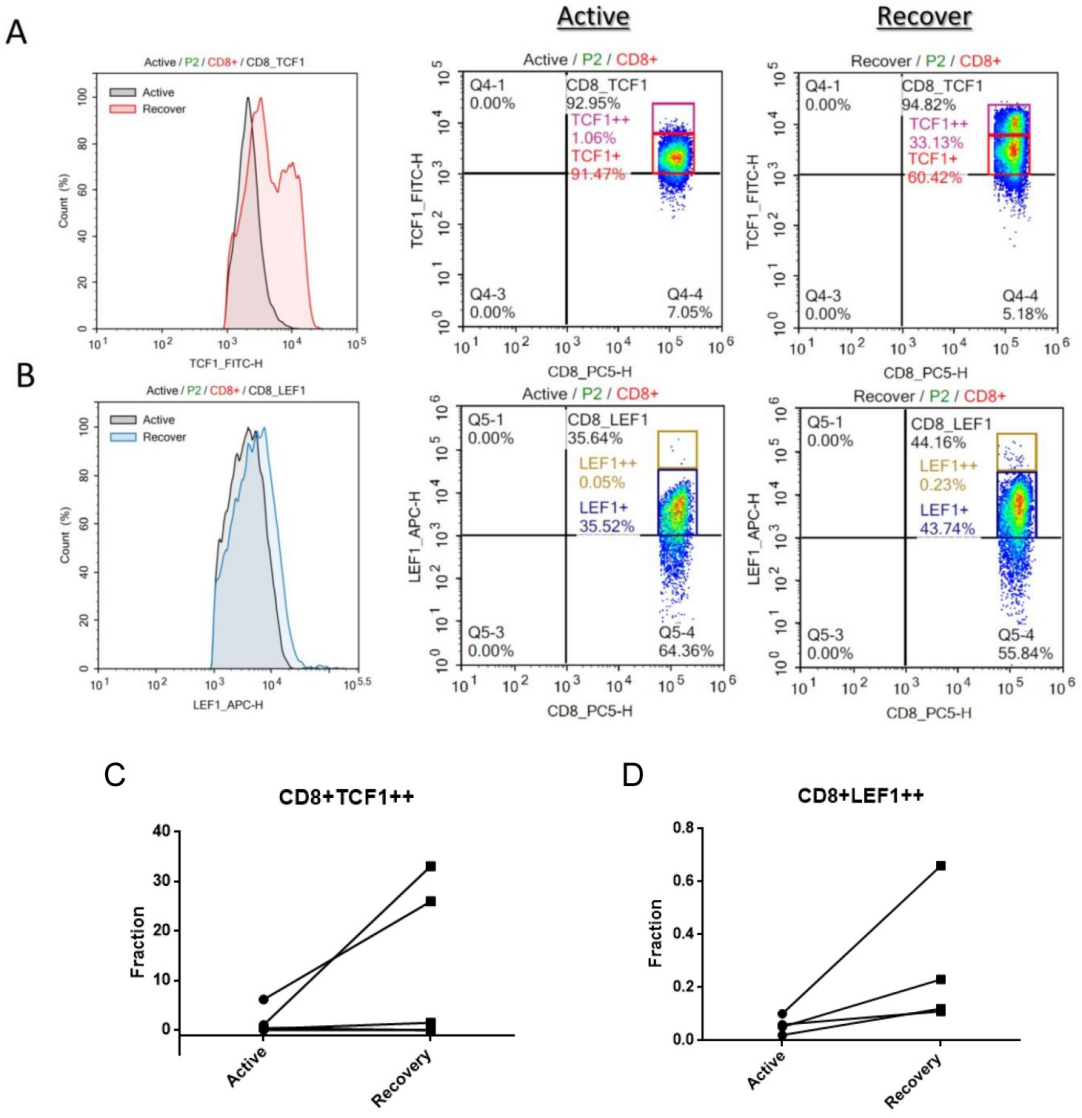
** Intracellular LEF1 and TCF1 protein expression in CD8 T cell subsets of SJS/TEN patients.** TCF1 and LEF1 protein expression was detected in CD8 T cell subsets by intracellular FACS staining. Black line histograms indicate TCF1 (A) or LEF1 (B) staining at the active stage of disease course, and color line histograms represent staining at the recovered stage. Representative flow cytometry plots show the expression levels of TCF1 (A) or LEF1 (B) in peripheral CD8 T cells of SJS/TEN patients. A representative example from one SJS/TEN patient is shown from four individual SJS/TEN patients. (C-D) The fraction of TCF1^high^ CD8 T cells and LEF1^high^ CD8 T cells populations are increased at the recovery stage comparing with that at the active stage by intracellular FACS staining though no statically significance (p=0.18 and p=0.15, respectively).

To further determine whether Wnt signaling is fluctuated in CD8 T lymphocytes during the progression of SJS/TEN, we also examined LEF1 and TCF-1 protein expression in peripheral CD8 T cell subsets by intracellular FACS staining. As shown in Figure 2, a decrease in the protein levels of both TCF1 and LEF1 was detected in the CD8 T cells of SJS/TEN patients at the active stage of disease course as compared with that at recovered stage. The portion of TCF1^high^ and LEF1^high^ CD8 T cells at the active stage was smaller than that at the recovered stage, suggesting that Wnt signaling is repressed in the key effector cells involved in SJS/TEN.

### Endogenous Wnt inhibitors, DKK1 and WIF1, are increased in SJS/TEN

**Figure 3:**
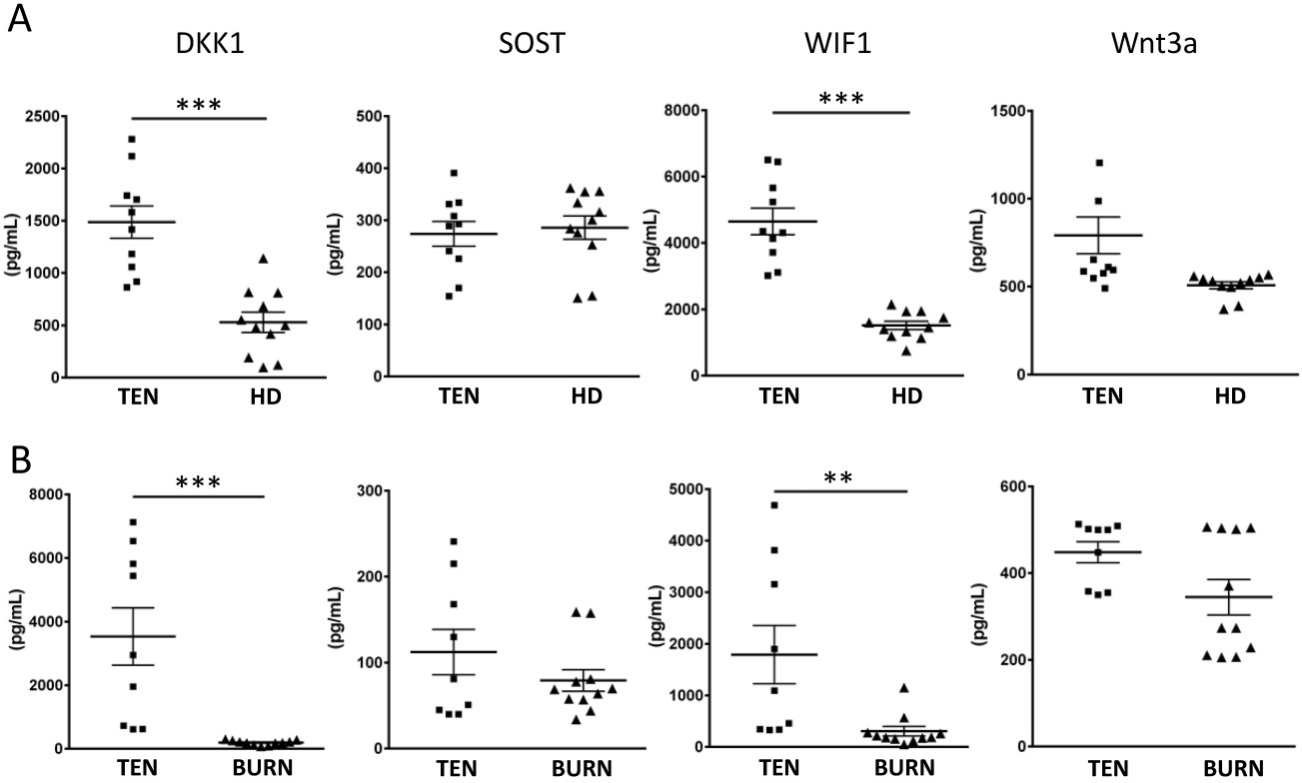
** Endogenous Wnt inhibitors associated with SJS/TEN.** (A) Serum levels of DKK1, SOST, WIF1, and Wnt3a were examined in 10 patients with SJS/TEN and 11 healthy donors (HD) by ELISA. (B) The levels of DKK1, SOST, WIF1, and Wnt3a in blister fluid of 9 TEN patients and 11 burn patients were measured by ELISA. * *p*< 0.05; ** *p*< 0.01; *** *p*< 0.001; two-sided Student's t-test.

Given the sufficiency of LEF1/TCF1 in modulating T cell responses during drug hypersensitivity reaction, a critical question is how this pathway is regulated in SJS/TEN. Many secreted proteins are known to serve as endogenous regulators of Wnt signaling (e.g. DKK1, SOST, WIF1, and Wnt3a). We, thus, tested whether these endogenous regulators of Wnt signaling are regulated in SJS/TEN by measuring the levels of DKK1, SOST, WIF1, and Wnt3a in the sera from SJS/TEN patients and normal subjects. We found that two endogenous Wnt inhibitors, DKK1 and WIF1, were elevated in the sera of SJS/TEN compared to health donors (Figure 3A). Furthermore, the levels of these endogenous Wnt regulators in the blister fluid from the SJS/TEN and burn patients were also evaluated. The levels of DKK1 and WIF1 in blister bluid of SJS/TEN patients were higher than that in blister fluid of burn patients (Figure 3B). These findings in part provide clues for regulation of Wnt signaling in SJS/TEN.

### IL-15 contributes to the attenuation of Wnt signaling in CD8+ T cells

**Figure 4:**
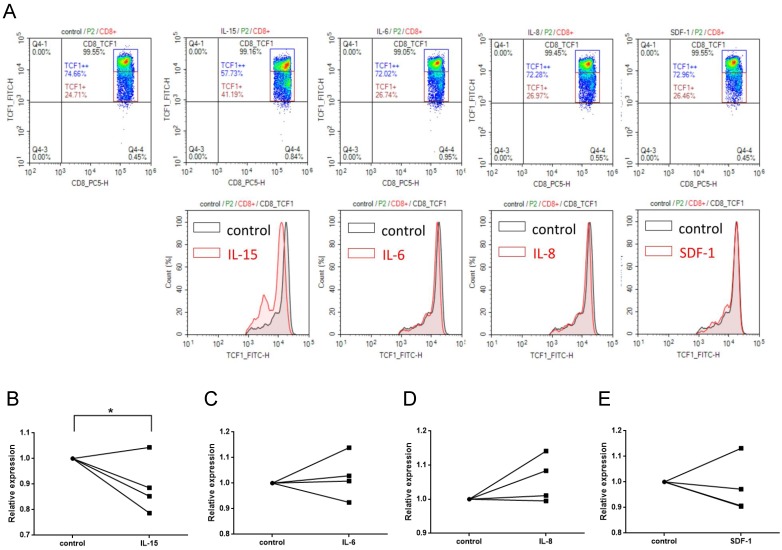
** IL-15 contributes to the attenuation of Wnt signaling in CD8+ T cells.** (A) TCF1 protein expression was detected in CD8+ T cell subsets by intracellular FACS staining after PBMCs were treated with cytokines indicated for 24 hr. Representative flow cytometry plots were shown from one of three independent experiments. (B-E) We observed a significant decrease in the portion of TCF1^high^ CD8 T cells in PBMCs from normal subjects that were treated with IL-15, but not with IL-6, IL-8, or SDF-1(stromal cell-derived factor-1, used as a non-SJS-related cytokine). * *p*< 0.05; two-sided Student's t-test.

The crosstalk between pro-inflammatory cytokines (e.g. IL-6, IL-8, IL-15, and TNF-a) and Wnt signaling pathway had been demonstrated 31, 32. We have recently shown that serum levels of several cytokines and cytolytic proteins were elevated in SJS/TEN 33. Of note, among these cytokines regulated in SJS/TEN, IL-15 was shown to be associated with the clinical severity and death of this condition 33. Here, we demonstrated that a decrease in the portion of TCF1^high^ CD8 T cells was observed as PBMCs from normal subjects were treated with IL-15 for 24 hours but not with IL-6, IL-8, or SDF-1(stromal cell-derived factor-1, used as a non-SJS-related cytokine) (Figure 4), revealing an impact of IL-15 on altering the activation of Wnt signaling in primary CD8+ T cells.

### Drug-mediated TCR activation attenuates the Wnt signal transduction

**Figure 5:**
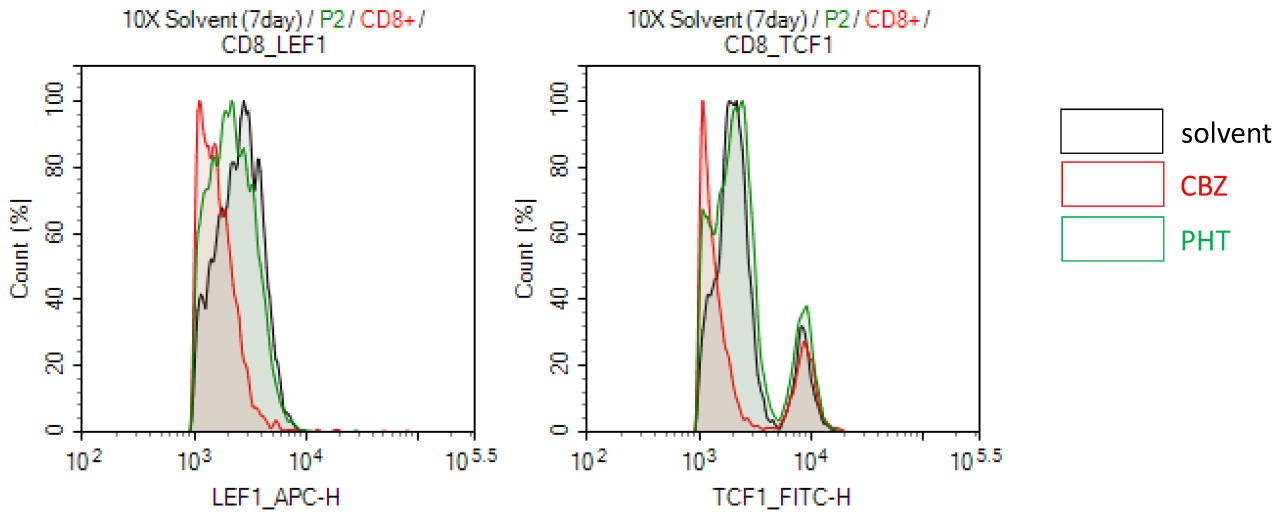
** Drug-specific TCR engagement attenuates the Wnt signal transduction in SJS/TEN.** PBMCs of a carbamazepine (CBZ)-induced SJS/TEN patient were stimulated with the culprit drug (CBZ) or a tolerant drug (phenytoin, PHT) for 7 days. Levels of LEF1 and TCF1 protein expression were detected in CD8+ T cell subsets by intracellular FACS staining. Representative flow cytometry plots were shown from one of five independent experiments.

Specific HLA types is highly associated with specific drug induced-SJS/TEN, which implies that specific HLA molecules may have higher binding affinity for specific drug antigens and present the drug antigens to specific TCR, further causing T cell activation and adverse responses 13. Thus, we further test whether drug-mediated engagement of TCR affects Wnt signaling in SJS/TEN. For example, PBMCs of a carbamazepine (CBZ)-induced SJS patient were stimulated with the culprit drug (CBZ) or a non-causative drug (phenytoin, PHT) for 7 days. The expression levels of genes related to Wnt signaling involved in T cell function were then examined in CD8+ T cells. We observed that the treatment of CBZ but not PHT attenuated the expression levels of LEF1 and TCF-1 proteins in CD8+ T cells of CBZ-induced SJS patients (Figure 5), suggesting that specific TCR engagement may dampen Wnt signaling in SJS/TEN.

### Wnt signaling is functionally implicated in the pathobiology of SJS/TEN

**Figure 6:**
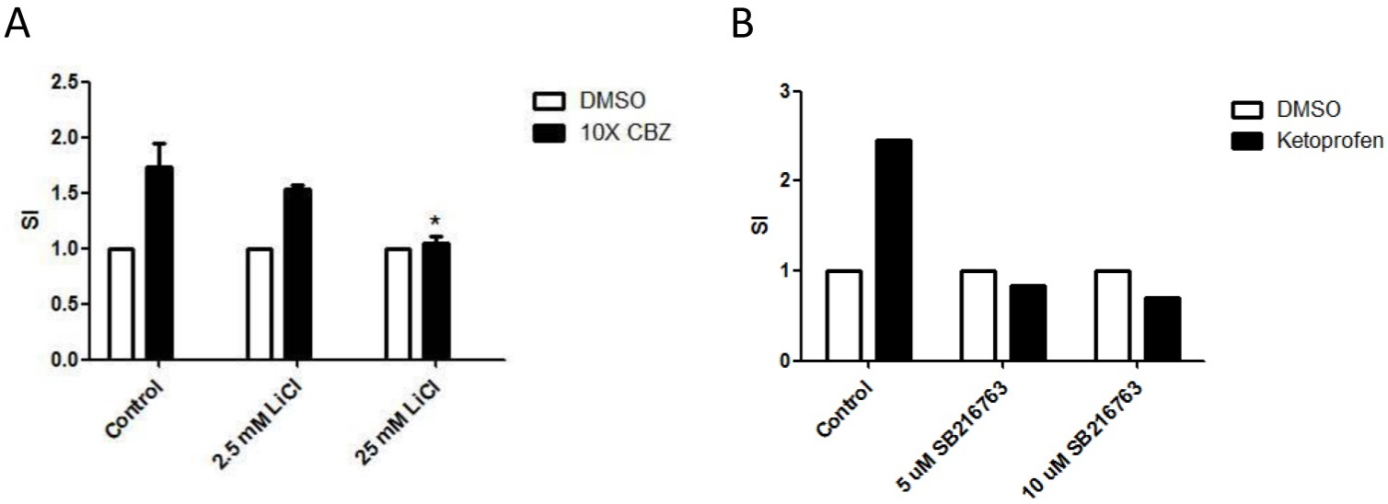
** Activation of Wnt signaling attenuates activation of drug-specific T cells.** PBMCs of a carbamazepine (CBZ)- (A) or Ketoprofen-sensitive (B) SJS patient were obtained from the heparinized blood by density centrifugation on Ficoll-Hypaque and stimulated with and without the culprit drugs in the absence or presence of different concentrations of Wnt signaling activator including LiCl (A) or SB216763 (B). After 7 days, the stimulation index (SI) will be calculated by the level of secreted granulysin of stimulated to unstimulated cultures. * *p*< 0.05; two sided Student's t-test.

Previous studies have revealed critical requirements for Wnt signaling in regulation of mature T cell responses 19. The effect of Wnt signaling on drug-specific activation of T cells in SJS/TEN, therefore, was examined. To manipulate the Wnt signal pathway, LiCl and SB-216763, two GSK3 inhibitors, were used to pretreat the drug-specific T cell clones to activate the Wnt signaling in effector cells. Consistently, the presence of LiCl or SB-216763 repressed the activation of drug-specific T cell in SJS/TEN (Figure 6A and 6B), indicating a functional involvement of this signal pathway in the pathomechanism of SJS/TEN.

## Discussion

CD8+ T cells and TCR function play a key role in the development of SJS/TEN, which is characterized as widespread epidermal and mucosal necrosis 34. Cytotoxic CD8 T cells that produce cytotoxic molecules (such as granulysin and granzyme B) to cause extensive keratinocyte death, are enriched in blister fluid samples from the skin lesions of patients with SJS/TEN 27, 35. These cytotoxic cells mediate the disease pathogenesis and are correlated with the disease severity and mortality. In this study, we showed that Wnt signaling components, including LEF1, TCF1, and LRP6, were downregulated in SJS/TEN patients, while the serum levels of endogenous Wnt inhibitors, DKK1 and WIF1, were elevated. These findings revealed that canonical Wnt signaling is regulated in SJS/TEN. Further assays demonstrated that engagement of the TCR by the specific causative drug and the treatment of a biomarker of SJS/TEN, IL-15 *in vitro* led to the downregulation of LEF-1 and TCF-1 expression in CD8+ T cells from SJS/TEN patients. Manipulation of Wnt signaling by adding the Wnt activators attenuated *ex vivo* activation of drug-specific T cells from SJS/TEN patients. Our data here, for the first time, suggest a functional involvement of Wnt signaling in the pathomechanism of SJS/TEN.

The canonical Wnt signaling transduction cascade is an evolutionarily conserved and multi-functional pathway and plays a crucial role in T cell development, differentiation, and functionality 36. Upon the activation of the Wnt signal pathway, naive CD8+ T cells undergo clonal expansion and further differentiate into effector and memory precursors. The Wnt signal pathway negatively regulates differentiated effector CD8+ T cells and positively upregulates memory precursor CD8+ T cells through TCF-1 alone or its combination with β-catenin 19. In addition, a role of Wnt signaling pathway in promoting generation of memory CD8+ T cells has been demonstrated through the observation that antigen-primed human memory CD8+ T cells expressed lower TCF-1 and LEF-1 than did naive T cells 22. Consistently, constitutive activation of the Wnt signaling pathway was found to reduce effector CD8+ T cell expansion 23, 24. Another study also confirmed these findings and further demonstrated that forced expression of stabilized β-catenin in naive T cells interfered with proximal TCR signaling 25. It is now clear that Wnt/β-catenin pathway is a key signaling pathway governing CD8+ T cell differentiation. However, little is known about the role of Wnt signaling pathway during the course of SJS/TEN.

The Wnt signaling pathway utilizes TCF-1/ LEF-1 transcription factors and β-catenin coactivator to achieve balanced regulation of its downstream gene expression upon the engagement of Wnt and its various receptors. Upon the activation of effector T cells, components of the Wnt signaling pathway, such as active form of β-catenin (dephosphorylated state), LEF1, TCF1, lipoprotein receptor-related protein-5 (LRP5, Wnt coreceptor), LRP6 (Wnt coreceptor), matrix metalloproteinase 2 (MMP2, Wnt target gene), and MMP9 (Wnt target gene) are differentially expressed 29, 37. In this study, we found that genes encoding Wnt signal components, including LEF1, TCF1, and LRP6, were downregulated in PBMC as well as in blistering cells from SJS/TEN as compared with normal subjects. Our study further demonstrated decreased intracellular LEF1 and TCF1 protein expression in CD8 T cell subsets of SJS/TEN patients, suggesting attenuation of Wnt signaling in CD8 T cells in SJS/TEN. We have previously shown that a number of cytokines was upregulated in SJS/TEN patients, of which IL-15 was significantly correlated with the disease severity and mortality of SJS/TEN 33. Here, we found that Wnt singals was attenuated specifically by IL-15 but not IL-6 or IL-8. In addition to the impact from SJS-related cytokines, Wnt signaling was dampened in CD8 T cells from SJS patients by TCR engagement with the culprit drug antigen. Moreover, we detected that endogenous Wnt inhibitors, DKK1 and WIF1, were elevated in the sera and bliser fluid of SJS/TEN compared to that from healthy donors and burn patients. These findings collectively provide clues for how Wnt signal pathway is regulated in the CD8 T cells in SJS/TEN. Furthermore, the presence of Wnt activators attenuated *ex vivo* activation of drug-specific T cells from SJS/TEN patients, indicating a functional involvement of Wnt signaling in the pathomechanism of SJS/TEN and a reasonable therapeutic implication.

In this study, we found that the LRP5 was not simultaneously downregulated compared with LRP6 in SJS/TEN though no statistical significance noticed. Active Wnt signaling is initiated by binding of a Wnt protein to its receptor (Frizzeld) and one of the coreceptors (LRP5 or LRP6). LRP5 is similar in sequence and structure to LRP6, and these two receptors have been proposed to function largely in the same contexts and signaling pathways, but there are differences between the two receptors. LRP5 usually can act as a gatekeeper for Wnt responses to enable Wnt pathway responsiveness 38. Comparing with LRP6, which exhibits strong signaling activity, LRP5 was reported to be much less active in cells with Wnt pathway activation 39. Here, we hypothesized that LRP6 could be the main coreceptor involved in the binding of a Wnt protein to its receptor (Frizzeld) and the coreceptors to generate a β-catenin/TCF Wnt signal. In addition, DKK1 and WIF1 were the most significant endogenous Wnt inhibitors comparing to SOST and Wnt3a involved in the reaction of SJS/TEN in this study. Previous literature has also shown that DKK1 (eliciting inhibition of LRP co-receptor function) and WIF1 (Wnt inhibitory factor 1) were both potential Wnt/β-catenin signaling therapeutic targets for T cell responses and cytokines mediated inflammatory autoimmune diseases.^40^ Further study was needed to understand the essential roles involved in the pathomechanism of SJS/TEN among these coreceptors and endogenous Wnt inhibitors, and it may provide evidence for the potential biological targets in the future.

The endogenous regulation in Wnt pathway in SJS/TEN. A previous study has demonstrated the endogenous proteins/ligands (including LEF-1 and TCF-1) are downregulated active Wnt signaling in effector T cells, which are the dominant immune cell involved in SJS/TEN in our study 23. These findings revealed consistent result between endogenous and exogenous ligand during the activation of effector T cells. In addition, a previous study had shown that endogenous LRP5 constitutively activates TCF/LEF-1 in the absence of Wnt 41. Another study also showed that removing the extracellular domain of both LRP5 and LRP6 could lead to constitutive activation of the intracellular domain. In the absence of exogenous Wnt 3a, full-length LRP6, but not LRP5, increased TCF/LEF-1 transcriptional activity, however both significantly potentiated Wnt 3a-induced TCF/LEF-1 activation.^42^ Moreover, the intracellular domains (membrane-anchored and cytosolic) of both LRP5 and LRP6 significantly increased TCF/LEF-1 activation in the absence of Wnt 3a, and potentiated the Wnt 3a-induced decrease in beta-catenin phosphorylation, increase in free beta-catenin levels and the increase in TCF/LEF-1 activity 42. These findings have demonstrated that LRP5 and LRP6 with and without the extracellular domain (whether or not they are membrane-anchored) could still facilitate Wnt 3a-induced TCF/LEF-1 activation. Taken together, the previous studies have shown the consistent results between exogenous and endogenous regulation and both played important roles in modulating Wnt signaling.

Active Wnt signaling also plays essential roles in the activation of other immune cells, including antigen presenting cells and Tregs, which are also known to be central to the pathomechansim of SJS/TEN 3, 18, 43. The canonical Wnt pathway was involved in suppressing dendritic cell activation and cross-priming of CD8+ T cell response 40. Activation of Wnt signaling could potentially increase the tolerogenicity of antigen presenting cells. On the other hand, Tregs from the acute-stage TEN patients profoundly are impaired in their suppressive function, resulting in more severe cytotoxic consequences 44. Interestingly, overexpression of stabilized beta-catenin in CD4+CD25+ Treg cells led to an increase of the survival of these Treg cells cells and could further induce an anergic phenotype in CD4+CD25- effector T cells, which favored the suppressive effect of Treg cells 21. In previous studies, Wnt signals may have the potential to restore Treg-mediated suppressive actions, which may be beneficial to reverse the overwhelming cytotoxic T lymphocyte mediated cytotoxicity 45. However, the role of Wnt signaling in Tregs function is still controversial since findings from another study showed that Wnt signals favored toward Th17 instead of Tregs lineage 46-48. It would be of interest and further investigations are necessary to delineate the exact role of Wnt signaling in the regulation of Tregs in SJS/TEN.

SJS/TEN are lethal severe cutaneous adverse reactions. Although the incidence has ameliorated by identifying the genetic predisposing factors, early diagnosis and prognosis monitoring for SJS/TEN remain a challenge for clinical physicians. Besides, no acceptable treatment guideline for this devastating condition highlights the inadequacy of the current therapeutic remedies. Our data here, for the first time, reveal that Wnt signaling is functionally involved in the immunopathogenesis of SJS/TEN. These findings suggest that enhancing the activity of Wnt pathway may be a potential strategy to modulate immunity in SJS/TEN and provide additional insight into therapeutic aspects of this devastating condition.

## Supplementary Material

Supplementary table S1.

## Figures and Tables

**Table 1:** Demographic and clinical characteristics of SJS/TEN patients

Demographic data	SJS/TEN (n=25)
**Age, years, mean ± SD (range)**	52.5 ± 18.7 (3-81)
**Sex ratio (M:F)**	9:16
**Skin (TBSA, %, mean ± SD (range))**	
Erythema	56.5± 26.7 (10-100)
Blister or detachment	30.9± 22.9 (1-80)
**Mucosa (n, (%))**	
Ocular	20 (80.0)
Oral	25 (100.0)
Genital	18 (72.0)
**Systemic Complications (n, (%))**	
Hepatitis ^a^	7 (28.0)
Acute kidney injury ^b^	6 (24.0)
Gastrointestinal bleeding	6 (24.0)
Pneumonitis, pneumonia, or bronchiolitis obliterans	4 (16.0)
**Blood dyscrasia (n, (%))**	
Eosinophilia ^c^	6 (24.0)
Leukocytosis ^d^	4 (16.0)
Leukopenia ^e^	9 (36.0)
Atypical lymphocytosis ^f^	16 (64.0)
Thrombocytopenia^g^	7 (28.0)
**Corneal ulcer or symblepharon (n, (%))**	5 (20.0)
**Fever ^h^ (n, (%))**	13 (42.0)
**Mortality (n, (%))**	5 (20.0)
**Underlying disease, n**	
Malignancy	2
Chronic kidney disease	4
Chronic liver disorder	3
Diabetes	3
Hypertension	9
Gouty	5
Epilepsy/Neuralgia	5
Rheumatoid arthritis	1
**Culprit drugs, n**	
Anticonvulsants ^i^	8
Antibiotics^ j^	7
Allopurinol	4
Chlorzoxazone	1
Dapsone	1
NSAIDs	1
PPI	1
Sulfasalazine	2

SD, standard deviation; SJS, Stevens-Johnson syndrome; TEN, toxic epidermal necrolysis; TBSA, total body surface area; NSAIDs, Non-Steroidal Anti-Inflammatory Drug; PPI, proton pump inhibitor. ^a^ Values were 2 times greater than normal for glutamic-oxaloacetic transaminase, glutamic-pyruvic transaminase, or total bilirubin. ^b^ The value of creatinine was 1.5 times greater than the normal value range (0.4-1.5 mg%) after drug intake. ^c^ Eosinophils > 0.7 × 10^9^/L, or > 5%, or >10% if leukocyte < 4.0 × 10^9^/L. ^d^ The leukocyte count was greater than 11,000/μL. ^e^ The leukocyte count was less than 3,500/μL. ^f^ Abnormal lymphocytes present in blood. ^g^ The platelet count was lower than 150,000/μL. ^h^ Patients had a body temperature greater than 38.5°C. ^i^ Carbamazepine, lamotrigine, phenobarbital, and phenytoin. ^i^ Cephalexin, flomocef, moxifloxacin, levofloxacin, and vancomycin.

## Abbreviations

SJS: Stevens-Johnson syndrome
TEN: toxic epidermal necrolysis
SCORTEN: severity of illness score of toxic epidermal necrolysis
RegiSCAR: Registry of Severe Cutaneous Adverse Reactions
BSA: body surface area
CTL: cytotoxic T lymphocyte
NK: natural killer
MHC: major histocompatibility complex
PBMC: peripheral blood mononuclear cell
LEF-1: lymphoid enhancer binding factor 1
LTT: lymphocyte transformation test
MPE: maculopapular exanthema
DRESS: drug reaction with eosinophilia and systemic symptoms
