# Supplementary material for: Attenuation of Wnt/β-catenin signaling in patients with Stevens-Johnson syndrome and toxic epidermal necrolysis
Source: Int J Biol Sci. 2020 Jan 1;16(2):353–64. doi: 10.7150/ijbs.32331 (PMC6949146; doi:10.7150/ijbs.32331)
Supplement: Supplementary file 1 — Supplementary table S1. [file ijbsv16p0353s1.pdf]

Supplement Table 1. Demographic data and clinical characteristics of control groups from burn injury patients and healthy donors.

Demographic data	Burn injury patients (n=11)	Healthy donors (n=11)
**Age, years, mean ± SD (range)**	42.5±11.4 (20-68)	43.5±12.7 (23-67)
**Sex ratio (M:F)**	4:7	5:6
**Skin (TBSA, %, mean ± SD (range))**		
Erythema	54.2±31.0 (3–50)	-
Blister or detachment	26.6±17.5 (5–90)	-
**Mortality (n, (%))**	0	-
**Underlying disease, n**		
Malignancy	0	0
Chronic kidney disease	1	0
Chronic liver disorder	0	0
Diabetes	1	0
Hypertension	1	0
Gouty	0	0
Epilepsy/Neuralgia	0	0
Rheumatoid arthritis	0	0
